# Evaluating TCFD reporting—A new application of zero-shot analysis to climate-related financial disclosures

**DOI:** 10.1371/journal.pone.0288052

**Published:** 2023-11-02

**Authors:** Alix Auzepy, Elena Tönjes, David Lenz, Christoph Funk

**Affiliations:** 1 Chair of Banking & Finance, Justus-Liebig-University, Giessen, Germany; 2 Department of Econometrics and Statistics, Justus-Liebig-University, Giessen, Germany; 3 istari.ai, Mannheim, Germany; 4 Centre for International Development and Environmental Research (ZEU), Justus-Liebig-University, Giessen, Germany; Zayed University, UNITED ARAB EMIRATES

## Abstract

We examine climate-related disclosures in a large sample of reports published by banks that officially endorsed the recommendations of the Task Force for Climate-related Financial Disclosures (TCFD). In doing so, we introduce a new application of the zero-shot text classification. By developing a set of fine-grained TCFD labels, we show that zero-shot analysis is a useful tool for classifying climate-related disclosures without further model training. Overall, our findings indicate that corporate climate-related disclosures increased after the launch of the TCFD recommendations and following individual endorsements. However, there are marked differences in the extent of reporting by recommended disclosure topic, suggesting that some recommendations have not yet been fully met. Our findings yield important conclusions for the design of climate-related disclosure frameworks.

## Introduction

Published in 2017, the recommendations of the Financial Stability Board’s Task Force on Climate-related Financial Disclosures (TCFD) have been described by the Government of the United Kingdom (UK) as “one of the most effective frameworks for companies to analyse, understand and ultimately disclose climate-related financial information” [1].

The TCFD recommendations, which have been formally endorsed by more than 4,000 companies worldwide to date, are a set of voluntary disclosure guidelines aimed at providing consistent climate-related information to investors and other key company stakeholders [2]. Compared to other reporting frameworks (e.g. Carbon Disclosure Project, Global Reporting Initiative), a particular focus of the TCFD recommendations is on disclosing information about the integration of climate-related considerations into risk management, control structures and strategic aspects of business operations [3]. Overall, the recommendations are organized into four primary disclosure categories (Governance, Strategy, Risk Management, Metrics and Targets) and eleven corresponding recommended disclosures associated with each category. For an overview of the TCFD recommendations, see Table 1.

**Table 1:** The TCFD disclosure categories and underlying recommended disclosures. Source: [15].

**Broad disclosure categories:**			
**Governance**	**Strategy**	**Risk Management**	**Metrics and Targets**
Disclose the organization’s governance around climate-related risks and opportunities.	Disclose the actual and potential impacts of climate-related risks and opportunities on the organization’s businesses, strategy, and financial planning where such information is material.	Disclose how the organization identifies, assesses, and manages climate-related risks.	Disclose the metrics and targets used to assess and manage relevant climate-related risks and opportunities where such information is material.
**Underlying Recommended Disclosures:**			
a) Describe the board’s oversight of climate-related risks and opportunities.	a) Describe the climate-related risks and opportunities the organization has identified over the short, medium, and long term.	a) Describe the organization’s processes for identifying and assessing climate-related risks.	a) Disclose the metrics used by the organization to assess climate-related risks and opportunities in line with its strategy and risk management process.
b) Describe management’s role in assessing and managing climate-related risks and opportunities.	b) Describe the impact of climate-related risks and opportunities on the organization’s businesses, strategy, and financial planning.	b) Describe the organization’s processes for managing climate-related risks.	b) Disclose Scope 1, Scope 2, and, if appropriate, Scope 3 greenhouse gas (GHG) emissions, and the related risks.
	c) Describe the resilience of the organization’s strategy, taking into consideration different climaterelated scenarios, including a 2°C or lower scenario.	c) Describe how processes for identifying, assessing, and managing climaterelated risks are integrated into the organization’s overall risk management.	c) Describe the targets used by the organization to manage climate-related risks and opportunities and performance against targets.

Since reliable information on climate-related exposure is critical for making informed investment decisions and appropriately pricing risks, an increasing number of investors have been exerting pressure on companies to issue reports that include comprehensive climate-related disclosures [4]. In addition, several countries, including the UK and Switzerland, have taken steps to make TCFD reporting mandatory for large companies in their jurisdictions. From a company perspective, the disclosure of climate-related information often signals awareness and preparedness for climate-related issues, while the absence of disclosure may, on the contrary, indicate that such issues are not being sufficiently addressed by the company [5, 6]. Illustrating these arguments, Jung et al. [7] and Subramaniam et al. [8] show that firms are more likely to integrate risks associated with climate change into their overall risk management when such firms also disclose climate-related information.

Against this background, it is surprising to find that research on climate-related disclosures remains sparse. More importantly, prior studies on the TCFD recommendations mostly focus on the quantity of information disclosed at the aggregate TCFD category level, and have left an analysis of the reported content within each category largely untouched. A more in-depth analysis within each of the 4 TCFD pillars is essential, as the quality and financial materiality of the disclosed information may vary depending on the industry [2]. For example, Bingler et al. [6] investigate climate-related disclosures based on the four core TCFD disclosure categories from 2015 to 2020. Their findings reveal that firms selectively disclose climate-related information, mainly concentrating on Governance and Risk Management, which the authors regard as the least material categories. Similarly, Ding et al. [9] analyze how carbon emissions affect voluntary climate-related disclosures at the TCFD category level. Their results show that firms with higher levels of carbon emissions disclose more climate-related information. Specifically, they report a positive relationship between carbon emissions and disclosures at the category level for strategy, risk management and metrics and targets. Finally, Friederich et al. [2] analyze the types of climate risks (physical or transition) reported in corporate annual reports. Their study provides evidence that disclosures related to transition risks have experienced a more pronounced increase compared to disclosures concerning physical risks, which are still lagging behind. While the authors take a more granular approach with regard to the materiality of climate-related disclosures, their findings do not encompass all the recommended TCFD disclosures, as climate risks make up only a small portion of the multifaceted issues to be addressed in TCFD reporting.

A shared characteristic of these studies is to rely on computerized textual analysis techniques, such as natural language processing (NLP), for the evaluation of climate-related disclosures. An important contribution in this regard is the work by Bingler et al. [6], who introduced “ClimateBERT”, a BERT model specifically fine-tuned to identify climate-related information within company disclosures based on the broad TCFD categories. Similarly, Friederich et al. [2] employ multiple pretrained BERT-related models, including DistilBERT and RoBERTa, to examine references to climate risks in company reports. Overall, these studies present mixed results regarding the effectiveness of climate-related disclosures in delivering high-quality and material information, primarily due to challenges such as greenwashing, a lack of transparency, and insufficient availability of quantitative data [10].

Motivated by the importance of a detailed and comprehensive analysis of climate-related disclosures not only at the broad TCFD category level, but also within each individual category, we contribute to the literature in two ways. First, we provide novel insights into the state of disclosures related to the TCFD recommendations by examining a sample of 3,335 reports published by TCFD-supporting banks between 2010 and 2021. Our focus on the banking industry is due to two key factors. On the one hand, it aligns with the fact that the TCFD recommendations primarily target financial institutions. On the other hand, it acknowledges the varying materiality of sustainability-related information across different industries. Within the financial sector, the identification and reporting of carbon-related asset concentrations hold particular importance due to the impact of climate change on credit risk and the potential risks associated with stranded assets [11, 12]. Thus, we build a sample of TCFD-supporting banks by retrieving all banks that have publicly declared their support to TCFD and are listed as official supporters on TCFD’s website. To leverage information on the TCFD recommendations beyond the 4 core disclosure categories, we develop a set of 14 fine-grained labels that are designed to capture the most central aspects of the TCFD recommendations for banks using similar semantics.

Second, we contribute to the literature on the use of NLP in the context of climate-related financial disclosures by introducing the zero-shot text classification as a new method for systematic and automated extraction of textual information from large amounts of reports including climate-related data. The zero-shot approach offers a critical advantage over other language models as it allows for sentence classification based on labels for which it has received no specific prior training. Our model relies on a multi-label approach and assigns probabilities (ranging from 0 to 100%) to each extracted text sequence in our sample of 3,335 reports. These probabilities represent the likelihood that a given text sequence aligns with a specific label. A higher probability suggests that the semantics of a text sequence match the semantics of the corresponding label, indicating that the sequence addresses the topic specified by the label. Thus, when a text sequence explicitly and precisely discusses a topic related to a label, it receives a higher label probability through the zero-shot text classification. In our analyses, we interpret higher label probabilities as proxys of disclosure quality. Furthermore, a higher label probability can also serve as an indication of the extent of disclosure on a particular topic, as a more detailed coverage of the topic is likely to result in a higher probability. As our method does not require any labeled training data, it also does not impose any restrictions on the number of labels (or classes), which allows us to perform a more detailed analysis of the underlying TCFD recommended disclosures. Additionally, the TCFD recommendations are well-suited for the zero-shot analysis, as they provide us with an already-existing framework and semantics [9].

In contrast, a weakness of ClimateBERT [13] and, more generally, of algorithms trained to identify and classify climate-related content is that such models require an extensive training set of human-labeled sentences. Manual labeling of sentences is not only time-consuming, but can also be error-prone. Therefore, for quality and consistency reasons, highly-trained and specialized “labelers” are required, which can also make the labeling process costly. Furthermore, the more classes (or categories of labels) to be included into the classification scheme of the model, the more labeled data is needed to ensure that each class comes with a reasonable amount of examples attached to it, which can be a limiting factor in some scenarios.

Our paper yields the following sets of findings. First, we investigate the level of disclosure within our sample of TCFD-supporting banks at the broad category level. Specifically, we develop two different types of labels: “general labels” that cover topics that are not specifically related to climate, as well as “climate-related labels” that correspond to the broad TCFD categories (i.e., climate-related governance, climate-related strategy, climate-related risk management and climate-related metrics and targets). We find that the mean probabilities relating to the general labels remain stable over the period from 2010 to 2021, while we observe an increase in all of the probabilities for the climate-related labels at category level over the same time period. In particular, we report that the disclosures related to “climate-related strategy” and “climate-related metrics and targets” grew particularly dynamically, reaching mean probabilities of up to 22% and 20% respectively in 2021, compared to 12% in 2010. Overall, this suggests an increased attention and priority given to the development of climate-related business strategies with corresponding targets among TCFD-supporting banks.

As a next step, we analyze the mean probabilities associated with our fine-grained labels, which cover the underlying recommended disclosures. This approach enables us to provide a more nuanced assessment of reporting quality beyond each broad TCFD category. Our results indicate substantial variation and notable gaps in reporting. In the strategy area, which is the most comprehensive category and contains several specific recommended disclosures for banks, we find that label probabilities are particularly low for disclosures related to financing and investment activities for carbon-intensive industries such as the fossil fuel industry. Similarly, in comparison to other strategy-related topics, TCFD-supporting banks exhibit a reduced likelihood of explicitly addressing the use of climate-related scenario models in their reporting. Under metrics and targets, we find that the incorporation of climate-related performance metrics into remuneration policies is associated with a lower label probability compared to labels related to carbon footprints and emissions reduction targets. In the governance area, TCFD-supporting banks demonstrate comparable levels of disclosure regarding the board’s responsibility in overseeing climate-related issues and the management’s role in assessing and managing such matters.

Third, as joining TCFD necessitates internal capacity and preparation (e.g., due to data collection), not all of the banks joined directly in 2017. We follow the approach in Bingler et al. [6] and investigate whether climate-related disclosures increased after the official launch of the TCFD recommendations in 2017 and after banks individually endorsed the TCFD recommendations. Overall, we find that the individual support of the TCFD recommendations goes along with an increase in climate-related reporting, which is statistically significant but economically modest. In terms of magnitude, we find a total average increase of 2.72% across all labels, which is in line with Bingler et al. [6] who report an increase of approximately 2.2 percentage points. Examining the disclosures of the banks that became supporters after the official TCFD launch, the most notable differences are observed in the Metrics and Targets category, and pertain to carbon footprints as well as emissions reduction targets. Consequently, our results indicate that TCFD-supporting banks enhance their level of disclosures relating to carbon emissions following their official TCFD endorsement, which is consistent with the findings of Ding et al. [9].

Altogether our findings are robust to various labels evaluation tests. We also examine whether our results are consistent with existing literature on the relationship between company size and CSR activities (e.g., Gillan et al. [14]). In line with our expectations, we report that larger banks exhibit higher disclosure probabilities compared to medium or small banks.

The remainder of this paper is organized as follows. First, we present our data, followed by our methods and model performance evaluation. Our results section is twofold. In the first part, we present the results of the zero-shot classification at the category level. In the second part, we analyze the results for the fine-grained labels covering the TCFD recommended disclosures. The results are summarized and discussed in the last section.

## Data

We apply the zero-shot classification to a sample of 3,335 hand-collected reports between 2010 and 2021. Due to the large differences between the homepages of the TCFD-supporting banks in our sample, a fully automated scraping approach is not possible. As a preliminary step, we extract the names of the TCFD-supporting banks from TCFD’s website, based on the industry categories “banks”, “central banks” and “capital markets”. After eliminating banks that could not be identified or lacked online annual reports, we are left with 188 TCFD-supporting banks. Table 2 presents the bank level data.

**Table 2:** Size and region of TCFD-supporting banks.

Region	Large	Medium	Small	∑
Asia Pacific	15	51	24	90
Europe	23	26	17	66
Latin America	0	4	3	7
Middle East & Africa	0	3	2	5
North America	9	8	3	20
∑	47	92	49	188

As a subsequent step, we proceed to categorize the banks in our sample based on two criteria: the region where their headquarters are located and their total asset size. In our analysis, we designate banks with total assets exceeding USD 500 billion as “large”, those with total assets ranging between USD 50 billion and USD 500 billion as “medium”, and banks with less than USD 50 billion as “small”. Interestingly, nearly half of our sample comprises banks from the Asia-Pacific region. European banks constitute around one-third of the sample, while North American banks represent approximately 10%. In terms of asset size, the majority of banks fall into the mid-sized category, with total assets ranging between USD 50 billion and USD 500 billion.

Next, we follow the approach in Bingler et al. [6] and collect available bank reports for the period 2010 to 2021 to capture textual data both before and after the publication of the TCFD recommendations in June 2017. The reports are classified according to the following categories: annual reports, CDP reports, corporate governance reports, integrated reports, remuneration reports, sustainability reports, and TCFD reports. Our analysis extends beyond relying solely on TCFD reports since most TCFD supporters do not publish standalone reports specifically dedicated to climate-related disclosures but rather integrate key information into their annual and sustainability reports. This aligns with the TCFD recommendations, which indicate that climate-related disclosures should be included in “mainstream (i.e., public) annual financial filings” [15]. The majority of reports in our sample consists of annual and sustainability reports.

After parsing the reports to ensure they are in a suitable raw text format for the zero-shot classification, we are left with a total sample of 3,335 bank reports, as illustrated in Table 3. In comparison to prior TCFD-related studies, the zero-shot allows us to examine a comparatively large sample of reports. As a comparison, Ding et al. [9] apply computerized textual analysis to a sample of 140 reports from TCFD signatories, while Demaria and Rigot [16] examine the reference documents of a sample of 40 French firms between 2015 and 2018.

**Table 3:** Sample composition.

Report Category	Number of reports	Average pages	Average number of sentences
Annual Report	1,869	207.98	2,711.21
CDP Report	75	63.43	699.79
Corporate Governance Report	148	69.44	1,014.25
Integrated Report	183	163.98	2,354.95
Remuneration Report	83	36.88	494.42
Sustainability Report	896	81.01	1,158.54
TCFD Report	81	36.68	544.37
	∑ = 3, 335	$\bar{x}=94.20$	$\bar{x}=1,282.50$

## Methodology

### Parsing PDFs

The reports utilized in our study are in PDF format. Extracting and converting textual information from PDF documents for subsequent analysis using NLP techniques is not as straightforward as working with text stored in CSV or TXT files. To address this challenge, we employ a layout-parsing model designed to detect and extract the actual text from PDF documents. In particular, we include the actual text from the reports and deliberately omit text from tables and graphs, which not only increases the quality of our data, but also saves computation time.

Our parsing model is based on Visual-Layout (VILA) groups introduced by Shen et al. [17]. VILA converts textual data into groups of tokens (text lines or blocks) and assigns a layout tag to these tokens. There are several variants of VILA, such as H-VILA (Visual Layout-guided Hierarchical Model) and I-VILA (Injecting Visual Layout Indicators). After conducting several trials, we select the H-VILA block variant trained on grotoap2 using the layoutLM model [18] since it delivers the best extraction and tokenization results. The output consists of the extracted text as groups of tokens together with the corresponding layout tags. Depending on the training set, the layout tags can be figures, body content, abstract and title. For our analysis, we keep the parts tagged as body content and abstract. In order to further improve our extraction results, we take further cleaning steps, which are summarized in Table 4.

**Table 4:** Cleaning steps of parsed raw texts.

Problem	Fix
Extra whitespaces	Replace extra whitespaces by single whitespace
Words separated by hyphen	Remove hyphen
Words separated by whitespace	Remove whitespace
Model parsed some ff, fi and if characters as one special character	Replace special double characters by normal characters
Whitespaces between word in sentence and punctuation	Remove whitespace
URLs in text which do not have any semantic meaning	Remove all urls from text
Parsed some sentences character by character with whitespace between them, i.e., S E N T E N C E instead of Sentence. Problem mostly occurred with figure subtexts	As most figure subtexts do not have important semantic meaning, we removed such single characters

### Zero-shot text classification

A widely used and important NLP task is text classification [19]. Text classification is used to organize and analyze very large amounts of textual data by assigning classes, or so-called “labels”, based on the topic of individual text sequences, which can consist of sentences, paragraphs, or entire pages. In general, text classification is carried out using neural network models, which can be as simple as basic neural networks or more sophisticated language models equipped with classification end-layers. These models (e.g., BERT or BART) undergo pre-training on extensive text data to acquire semantic understanding [10]. These pretrained models can subsequently be employed for various NLP tasks and fine-tuned for a specific task. The fine-tuning process involves combining different combinations of pre-trained language models (i.e., the base model) with task-specific end-layers.

Fine-tuning a base model can be accomplished by training it with labeled training data. As a result, the accuracy of the model often relies on the size and quality of the training data. However, creating a training set for sentence classification presents several drawbacks: First, the process of manually labeling large amounts of text is extremely time-consuming and requires significant human resources. Second, the labeling process must be repeated when classes need to be changed or new labels need to be added. Third, assigning the correct label to a sentence can be challenging even for humans, as certain sentences can be interpreted in different ways. Another drawback is the potential bias introduced by human labelers, making it difficult to obtain a representative training set [20]. Lastly, finding suitable training data poses a challenge since the training data cannot be the same as the data used for actual analysis. For instance, in the analysis of TCFD reports, the labeled reports used for model training cannot be reused. Moreover, due to the limited number of TCFD reports published by banks and the imperfect nature of TCFD reporting by companies, there is a limitation in acquiring high-quality training data.

**Fig 1:**
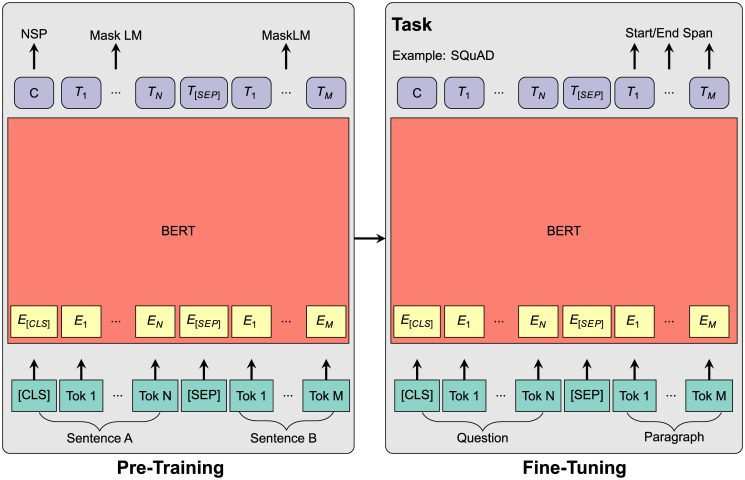
Model architecture overview. On the left hand side, the BART model is pre-trained on all English Wikipedia articles and the BooksCorpus dataset. By masking parts of sentences ([MASK]), the model is trained to learn the semantics and to predict the missing parts. The process is repeated for all sentences in the pre-training dataset. On the right hand side, the model is fine-tuned on the MNLI task and returns probabilities for entailment, contradiction and neutral, as shown on the left hand side. Source: Own representation.

In this paper, we address the aforementioned drawbacks by employing a zero-shot text classification model introduced by Davison [21]. The model is able to classify text sequences based on the semantics of the input sequences and the labels without further requiring additional training. A simplified structure of our model architecture is shown in Fig 1. To perform the zero-shot classification, we employ BART as a base model. The model has been pre-trained on approximately 160GB of text from the English Wikipedia and BookCorpus dataset to develop an understanding of textual semantics [10]. Given that the TCFD recommendations do not specifically focus on financial language but rather general semantics, we consider BART to be well-suited for our analysis. Additionally, since the reports in our sample are in English, we can leverage the fact that the model was pre-trained on a large English language corpus. Compared to other models like BERT, the BART model [22] utilizes a sequence-to-sequence translation architecture with bidirectional encoders (BERT) and a left-to-right autoregressive decoder (GPT model), combining the strengths of both. When used as a base model in conjunction with zero-shot text classification, BART demonstrates good performance results [21].

For our zero-shot classification, we employ a specific NLP task known as multi-natural language inference (MNLI) [21]. This approach embeds both the sentences of a text (sequence of words) and the labels themselves into a shared latent space. In this latent space, the proximity between the sentence and the label can be computed, indicating the probability of a match. The closer the label is to the sentence, the higher the probability that the label matches the sentence. In the context of zero-shot text classification, labels are therefore used to assess the probability that a text sequence addresses one (or more) labeled topics. While the zero-shot text classification yields probabilities from 0 to 100% for each label, the ClimateBERT model used in Bingler et al. [6] does not yield probabilities as output. Instead, it produces a binary output where one label is considered true and all others are false. For example, the classification output of one governance-related paragraph could be 1 for the label governance, and 0 for all other labels, i.e. 0 for strategy, 0 for risk Management etc. Compared to our approach, the authors measure the proportion of TCFD-related content in corporate reports by summing up the results for all labels and putting them in relation to the number of paragraphs [6].

In contrast, our text classification model treats text sequences as premises and labels as hypotheses. It tokenizes both the sentence and the label, leveraging the underlying language to embed them. These embedded representations are then processed through the pre-trained MNLI layer. The MNLI end-layer consists of a simple fully-connected neural network that produces a vector of logit scores for three possible outcomes: “neutral”, “contradiction” and “entailment” [21, 22]. Consequently, the hypothesis is evaluated against the premise, resulting in a classification of entailment, contradiction, or neutrality. The score for “neutral” is discarded and a softmax function is applied to the “contradiction” and “entailment” scores in order to be able to interpret them as a probability on a scale from 0 to 100%. In our analyses, the scores shown are for the “entailment” only. They can be interpreted as the probability that a text sequence matches a given label, or in other words, the probability that the given label is true. This fine-tuned end-layer can subsequently be used for zero-shot classification without any additional training.

Finally, due to the interconnected nature of the TCFD recommendations and the possibility for a sentence to align with multiple recommended disclosures simultaneously, we adopt a multi-label approach. Consequently, we do not constrain the zero-shot text classification to return probabilities that sum up to one, as in the single-label approach. Instead, we employ an approach where the model can assign probabilities ranging from 0 to 1 for each label (multi-label approach), accounting for the potential overlap between a sentence and multiple labels. As a result, the probabilities assigned to all labels for each sentence do not necessarily add up to one.

### Fine-grained TCFD labels

The TCFD has structured its recommendations along four core disclosure categories: Governance, Strategy, Risk Management, Metrics and Targets (see Table 1). Each category comprises two to three recommended disclosures, accompanied by detailed descriptions specifying the information to be included. Recognizing the varying materiality of information across industries, the TCFD has also developed additional guidance tailored to the financial sector, including banks, insurance companies, asset managers, and asset owners. In its guidance for banks, the TCFD emphasizes that climate-related disclosures from credit institutions should facilitate the identification of large concentrations of carbon-related assets and provide a better understanding of the financial system’s exposure to climate-related risks [15].

We proceed by creating a set of general labels covering each main category: **GO.1** *Climate-related Governance*, **ST.1** *Climate-related Strategy*, **RM.1** *Climate-related Risk Management*, **MT.1** *Climate-relate Metrics and Targets*. The inclusion of “climate-related” ensures that the zero-shot classification primarily captures sentences addressing climate-related topics. For a comprehensive overview of our fine-grained labels, please refer to Table 5. Next, we develop a set of targeted labels based on the recommended disclosures, their description, and the additional guidance provided for the financial sector. Under the governance pillar, we summarize the recommended disclosures, “describe the board’s oversight of climate-related risks and opportunities” and “describe management’s role in assessing and managing climate-related risks and opportunities” into two labels: **GO.1.1** *Board’s responsibility for overseeing climate-related issues* and **GO.1.2** *Executive management’s role related to the assessment and management of climate-related issues*. We include the terms “executive” and “strategic role” to highlight that these recommendations refer to the assignment of strategic responsibilities at executive management level. In addition, the TCFD often uses the expression “climate-related issues” to refer to climate-related risks and opportunities. Hence, we incorporate this expression in our labels.

**Table 5:** Overview of TCFD labels.

Governance	GO.1.	Climate-related Governance
	GO.1.1	Board’s responsibility for overseeing climate-related issues
	GO.1.2	Executive management’s strategic role related to the assessment and management of climate-related issues
Strategy	ST.1.	Climate-related Strategy
	ST.1.1	Climate-related transition risks such as policy, legal, technology, market and reputation risks emerging from climate change
	ST.1.2	Climate-related physical risks such as acute weather events and chronic shifts in weather patterns
	ST.1.3	Material financial impact of climate-related issues
	ST.1.4	Credit exposure to carbon-related assets
	ST.1.5	Financing and investment for carbon-intensive industries such as fossil fuel industry
	ST.1.6	Use of climate-related scenario models to analyse the impact of climate-related risks
	ST.1.7	Resilience of the bank’s strategy under different climate-related scenarios
Risk Management	RM.1.	Climate-related Risk Management
	RM.1.1	Processes to identify, assess and manage climate-related risks and integrate them into overall risk management
	RM.1.2	Relationship between climate-related risks and financial risks such as credit risk, market risk, liquidity risk and operational risk
Metrics & Targets	MT.1.	Climate-related metrics and targets
	MT.1.1	Carbon footprint, direct and indirect greenhouse gas emissions
	MT.1.2	Incorporation of climate-related performance metrics into remuneration policies
	MT.1.3	Emissions reduction and carbon neutrality targets

In the strategy area, we employ the labels **ST.1.1** *Climate-related transition risks such as policy, legal, technology, market and reputation risks emerging from climate change* and **ST.1.2** *Climate-related physical risks such as acute weather events and chronic shifts in weather patterns* to capture the recommended disclosure that states to “describe the climate-related risks and opportunities the organization has identified over the short, medium, and long-term”. Since the TCFD specifically recommends discussing examples of transition and physical risks, we distinguish between the two types of risks in our labels. Next, we turn to the second recommended disclosure in the strategy area, which is “describe the impact of climate-related risks and opportunities on the organization’s businesses, strategy, and financial planning”. Given that issues related to “businesses, strategy, and financial planning” are particularly difficult to summarize into one label, we rather focus on the first part related to the financial impact. We aim to capture whether financial institutions perceive climate change as having a significant financial impact on their operations. Based on this, we develop the label **ST.1.3** *Material financial impact of climate-related issues*. In addition, the TCFD encourages the description of climate-related scenarios if such scenarios are used [15]. Therefore, we create the label **ST.1.6** *Use of climate-related scenario models to analyze the impact of climate-related risks* to assess whether the banks in our sample conduct such analyses and disclose related information.

Additionally, the TCFD recommends banks to describe significant concentrations of credit exposure to carbon-related assets. This recommendation overlaps with a similar recommended disclosure in the metrics and targets category, which requires banks to “provide the amount and percentage of carbon-related assets relative to total assets as well as the amount of lending and other financing connected with climate-related opportunities”. Considering these recommendations, we create the labels **ST.1.4** *Credit exposure to carbon-related assets* and **ST.1.5** *Financing and investment for carbon-intensive industries such as fossil fuel industry*. Finally, we turn to the last recommended disclosure under the strategy category, “describe the resilience of the organization’s strategy, taking into consideration different climate-related scenarios, including a 2°C or lower scenario” and add the label **ST.1.7** *Resilience of the bank’s strategy under different climate-related scenarios*.

Within the risk management pillar, the TCFD advises to “describe the organization’s processes for identifying and assessing climate-related risks”, to “describe processes for managing climate-related risks”, and to “describe how processes for identifying, assessing, and managing climate-related risks are integrated into the organization’s overall risk management”. Using the label **RM.1.1** *Processes to identify, assess and manage climate-related risks and integrate them into overall risk management*, we combine the above recommended disclosures into one label. With respect to the additional guidance for banks, the TCFD advises to consider characterizing climate-related risks in the context of traditional banking industry risk categories such as credit risk, market risk, liquidity risks and operational risks. We therefore add the label **RM.1.2** *Relationship between climate-related risks and financial risks such as credit risk, market risk, liquidity risk and operational risk.*

Under the metrics and targets pillar, the TCFD recommends to “disclose the metrics used by the organization to assess climate-related risks and opportunities in line with its strategy and risk management process”. In this context, organizations are encouraged to provide key metrics used to measure and manage climate-related risks, and banks are particularly advised to disclose metrics employed to assess the impact of transitional and physical climate-related risks on their lending and other financial activities [15]. Additionally, banks are required to disclose “the amount and percentage of carbon-related assets relative to total assets as well as the amount of lending and other financing connected with climate-related opportunities”. These recommendations are closely related to the recommended disclosures under the strategy and risk management pillars, as they also involve the disclosure of metrics related to climate-related transition and physical risks and their impacts (e.g., labels **ST.1.1**, **ST.1.2**, and **ST.1.3**). Thus, we focus on a specific sub-element within the recommended disclosure, i.e. “where climate-related issues are material, organizations should consider describing whether and how related performance metrics are incorporated into remuneration policies”. We label this **MT.1.2** *Incorporation of climate-related performance metrics into remuneration policies*. This label aims to assess whether banks report on the inclusion of climate-related metrics in their compensation practices, reflecting the growing importance of sustainability-related performance measures in executive remuneration [15]. Next, in response to the recommended disclosure “disclose Scope 1, Scope 2, and, if appropriate, Scope 3 greenhouse gas (GHG) emissions, and the related risks”, we create the label **MT.1.1** *Carbon footprint, direct and indirect GHG*. Lastly, the TCFD recommends to “describe the targets used to manage climate-related risks and opportunities and performance against targets”. Since the TCFD encourages the disclosure of GHG emissions targets, as it also places great emphasis on gaining a better understanding of the concentrations of carbon-related assets in the financial sector, we focus on this particular type of target and create the label **MT.1.3** *Emissions reduction and carbon neutrality targets*.

## Label evaluation

The zero-shot text classification model used in our study does not require specific training with pre-labeled data. As a result, the model cannot be validated in the conventional manner of splitting a dataset into training and test sets and evaluating the model’s performance on the test set. Nonetheless, we can still conduct a series of evaluation tests to assess the text sequence recognition and classification performance of our zero-shot text classification model.

To create a dataset for evaluation, we manually collect sentences that align with the TCFD recommendations and assign them specific labels. These sentences are extracted from the TCFD good practice handbook [23, 24], which features examples of best practice disclosures selected by the TCFD. Additionally, we manually extract sentences from TCFD reports of companies outside the banking sector. We also incorporate sentences from the training repository provided by Webersinke et al. [13]. All of these sentences have been assigned a label by the authors, which is either “governance”, “strategy”, “risk management”, “metrics and targets” or “none”. We reassign one of our fine-grained labels to these text sequences.

**Fig 2:**
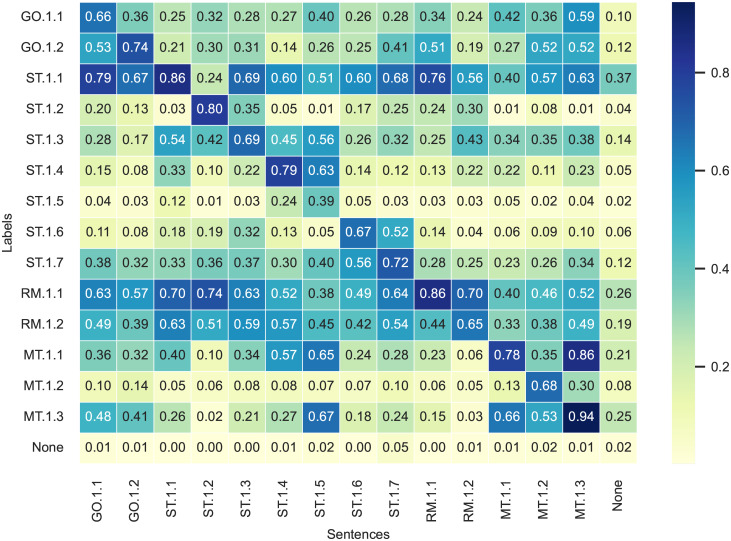
Label evaluation matrix based on test dataset. This matrix presents the results of the zero-shot text classification applied to our test dataset. The fine-grained TCFD labels are based on the recommended disclosures and the supplemental guidance for the financial sector.

In the first step, we apply the zero-shot classification to the dataset using a multi-label approach. This approach allows the zero-shot model to provide results for each label independently, acknowledging that a text sentence may correspond to multiple labels. Consequently, the results returned by the zero-shot classification for each label do not sum up to one. We utilize the fine-grained labels listed in Table 5. We also include the label “none” in the classification task. The purpose of this label is to capture non-climate-related text sequences, i.e., text sequences that do not fit any of our fine-grained labels. Including the “none” label ensures that the labels assigned by the zero-shot are based on the semantics of the text sequences rather than by pure chance. To assess the model’s performance with respect to the “none” label, we use sentences labeled as “none” in the aforementioned training repository, as well as additional sentences labeled as “none” by us. The results of the zero-shot text classification applied to the dataset are shown in Fig 2. The x-axis represents the manually annotated sentences, while the y-axis shows the results from our zero-shot analysis based on the climate-related labels. The results represent the mean probability returned by the zero-shot model that the sentences in a given column deal with the topic represented by the label in the corresponding row. Darker entries represent higher likelihoods classified by the model. In an optimal zero-shot text classification, the entries along the diagonal would be the darkest.

These results demonstrate that our model accurately assigns high probabilities to the appropriate labels. At the same time, we also observe that labels ST.1.1 (Climate-related transition risks), ST.1.3 (Material financial impact of climate-related issues), ST.1.7 (Resilience of the bank’s strategy), RM.1.1 (Processes to identify, assess and manage climate-related risks and integrate them into overall risk management), and RM 1.2 (Relationship between climate-related risks and financial risks) perform worse than other labels in terms of text sequence recognition. For example, both the groups of sentences that we labeled as risk management sequences as well as other sentence groups were assigned the labels RM.1.1 and RM1.2 with a probability of 60% or higher.

There could be several reasons for these results. First, the abstract nature of some topics covered may render them less suitable for zero-shot text classification. Terms such as “resilience” encompass a wide range of interpretations, posing challenges in precise reporting and potentially causing overlaps with multiple text sequences. This observation also brings attention to weaknesses in the design of the TCFD recommendations. Second, the TCFD recommendations often encompass closely interconnected themes, leading to situations where text sequences can align with multiple labels simultaneously. For example, the only label which does not have the highest probability for its own group of sentences is label MT.1.1 (Carbon footprint, direct and indirect greenhouse gas emissions), where a higher probability was assigned by the zero-shot to MT.1.3 (emissions reduction and carbon neutrality targets). Given the close relationship between these topics, it is unsurprising to observe high probabilities assigned to both sets of sentences. However, in order to maintain consistency with the semantics of the TCFD recommendations, we made a deliberate choice not to modify the labels.

The probabilities for the “none” label are consistently low across all sentences, indicating that the model does not simply label by chance, but rather incorporates the semantics of the labels in its classification. Sentences labeled as “none” received low probabilities for nearly all labels. The exception is label “climate-related transition risks” (ST.1.1), which has a slightly higher probability of 37% for the “none” sentences. This may be linked to the fact that this label encompasses many different types of risks, such as political and legal risks, technological risks, market risks, and reputation risks, all of which belong to the “transition risks” category. For some sentences describing these risks, the zero-shot classification may not directly identify the link to climate change. Furthermore, we also notice that more abstract labels such as RM.1.1 and RM.1.2 have higher values for “none” sentences as well.

In addition to our graphical analysis, we evaluate the model performance by examining the overall F1 scores and the individual F1 scores of our labels, as illustrated in Table 6. We evaluate the model based on test data previously used in Fig 2, focusing on our fine-grained labels. In contrast to the previous matrix, we calculate the F1-scores using a single-label approach, as we also performed a single-label approach by manually attributing a specific label to the text sequences in our dataset. Overall, our model obtains a micro F1 score of 0.60 and a macro F1 score of 0.57, which is satisfactory considering the presence of 14 classes. In addition, we observe that material financial impact of financial issues (ST.1.3) is the most challenging to identify (F1-score of 0.34) and the incorporation of climate-related performance metrics into remuneration policies (MT.1.2) the easiest (F1-score of 0.78). This discrepancy may be explained by the fact that financial material impact is a broader concept, and the task of identifying corresponding sentences may be more challenging, even for humans. We also observe that our governance labels exhibit a comparatively high precision. In contrast, the labels pertaining to transition risks (ST.1.1), the relationship between climate-related risks and financial risks (RM.1.2) and emissions-reduction targets (MT.1.3) display relatively lower precision, suggesting a higher number of false positives. Additionally, our labels exhibit relatively high recalls, with the exception of material financial impact of financial issues (ST.1.3.) and financing and investment for carbon-intensive industries (ST.1.5).

**Table 6:** Comparison of performance based on F1 scores.

Label	GO.1.1	GO.1.2	ST.1.1	ST.1.2	ST.1.3	ST.1.4	ST.1.5	ST.1.6	ST.1.7	RM.1.1	RM.1.2	MT.1.1	MT.1.2	MT.1.3
Recall	0.53	0.74	0.66	0.61	0.28	0.52	0.31	0.57	0.51	0.73	0.59	0.58	0.68	0.93
Precision	0.97	0.72	0.29	0.79	0.46	0.54	1.00	0.77	0.50	0.61	0.29	0.90	0.91	0.24
F1-Score	0.69	0.73	0.40	0.69	0.34	0.53	0.48	0.65	0.51	0.66	0.39	0.70	0.78	0.39

The overall performance scores are: Micro F1-score: 0.6029, Macro F1-score: 0.5668, Weighted F1-score: 0.6281.

Altogether, our zero-shot text classification does not appear to assign probabilities purely by chance. The TCFD recommendations appear to be intertwined, which is an argument for the multi-label approach we use. We also find that zero-shot classification yields better results when labels are based on well-delineated and precisely defined concepts.

## Results

### Climate-related disclosures by broad TCFD categories

The main objective of the TCFD recommendations is to guide companies in disclosing consistent and decision-useful information for key stakeholders [6]. These climate-related disclosures aim to reduce information asymmetry between reporting firms and stakeholders and demonstrate companies’ awareness of climate-related issues [4, 7, 9]. In a first step, we examine the probability that corporate reporting addresses climate-related issues and relates to one of the four main TCFD pillars. Table 7 presents the probabilities associated with the general labels “Governance”, “Strategy”, “Risk Management”, and “Metrics and Targets” as well as the probabilities for our labels “climate-related Governance” (GO.1), “climate-related Strategy” (ST.1), “climate-related Risk Management” (RM.1) and “climate-related Metrics and Targets” (MT.1), respectively. We intentionally include both types of labels to facilitate a comparison between disclosures on general topics and disclosures specifically related to climate-related matters.

**Table 7:** Mean of label probabilities at category level per financial year.

	2010	2011	2012	2013	2014	2015	2016	2017	2018	2019	2020	2021
Governance	0.31	0.31	0.30	0.30	0.31	0.31	0.31	0.31	0.31	0.31	0.32	0.32
GO.1	0.11	0.11	0.12	0.11	0.12	0.12	0.12	0.13	0.14	0.15	0.17	0.19
Strategy	0.40	0.40	0.40	0.40	0.39	0.40	0.39	0.40	0.39	0.40	0.40	0.40
ST.1	0.12	0.12	0.12	0.12	0.12	0.13	0.13	0.14	0.15	0.17	0.20	0.22
Risk management	0.23	0.23	0.24	0.23	0.24	0.23	0.23	0.23	0.23	0.23	0.24	0.24
RM.1	0.09	0.08	0.09	0.09	0.09	0.09	0.09	0.10	0.11	0.12	0.15	0.16
Metrics & Targets	0.31	0.31	0.31	0.31	0.31	0.32	0.31	0.32	0.32	0.32	0.33	0.34
MT.1	0.12	0.12	0.12	0.12	0.12	0.13	0.13	0.13	0.14	0.16	0.18	0.20

The table presents the mean of label probabilities (on a scale from 0 to 1) for the general and climate-related labels at category level based on the full sample of 3,355 reports.

Several observations can be made based on these results. First, the mean probabilities for the general labels, which do not specifically mention climate-related topics, are higher compared to the probabilities for the specific climate-related labels. For instance, the probability of reporting on “Governance” is consistently higher than the probability of reporting on “climate-related Governance” (GO.1) throughout the entire sample period. The same result holds true for the other main categories. This result is reasonable considering that our text sequences are extracted from various reports, including corporate governance reports and annual reports that cover a wide range of governance-related topics, not solely focused on climate-related governance. Thus, the zero-shot text classification model appears to effectively distinguish between climate-related and non-climate-related textual data.

Second, we observe that the mean probabilities associated with the general labels (without explicit mention of climate) remain stable over the sample period from 2010 to 2021, while there is an increase in all probabilities for the climate-related labels after 2017. Among the general labels, “Strategy” exhibits the highest mean probability compared to the others, maintaining a consistent probability of around 40% over time. In contrast, the probability of “climate-related Strategy” shows a dynamic increase, growing from 12% in 2010 to 22% in 2021. This indicates that the probability of text sequences in our sample relating to “climate-related Strategy” was only 12% in reports from 2010 but reaches 22% for reports published in 2021. In addition, we find that the label “Metrics and Targets” has the second highest mean probability over the sample period compared to the other labels with a mean probability between 31% and 34%. When examining the mean probability of “climate-related Metrics and Targets”, we find an increase from 12% in 2010 to 20% in 2021, surpassing the probabilities of “climate-related Governance” and “climate-related Risk Management” in 2021.

**Fig 3:**
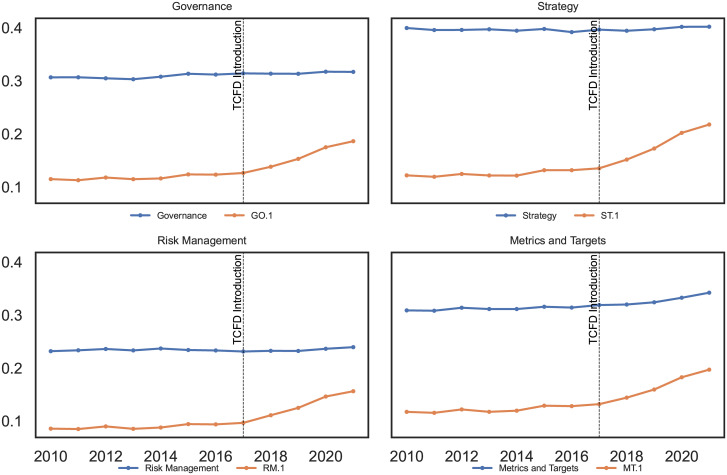
Climate-related disclosures by broad TCFD categories.

To further examine climate-related disclosures at the category level, we examine the trends in reporting before and after the publication of the TCFD recommendations in 2017. Fig 3 provides a visual representation of these trends for all four TCFD categories over the sample period. Specifically, the blue lines represent the label probabilities of the general labels, while the orange lines illustrate the probabilities of the climate-related category labels, denoted as GO.1, ST.1, RM.1 and MT.1 in Table 5. We observe an overall increase in all climate-related label probabilities, with a more pronounced change occurring around 2017.

### Climate-related disclosures by fine-grained TCFD labels

To more accurately assess the quality of climate-related disclosures, it is important to go beyond the quantity of reporting for each broad TCFD category and instead focus on examining corporate reporting on the specific recommended disclosures within each category. Therefore, as a next step, we consider the fine-grained labels that address specific topics related to the TCFD recommendations, rather than the climate-related category labels (GO.1, ST.1, RM.1, and MT.1). As highlighted earlier, the TCFD provides additional guidance for the financial sector, including banks, insurances, asset managers and asset owners [15]. The additional guidFance for banks particularly emphasizes disclosures related to strategy, risk management, and metrics and targets. We consider a higher label probability to serve as a proxy for disclosure quality. When a text sequence explicitly and accurately addresses a topic expressed in a label, it is associated with a higher label probability. A higher label probability also suggests a more comprehensive disclosure on a particular topic, as labeling is more likely to have a higher probability if a text sequence provides detailed information about the topic.

**Fig 4:**
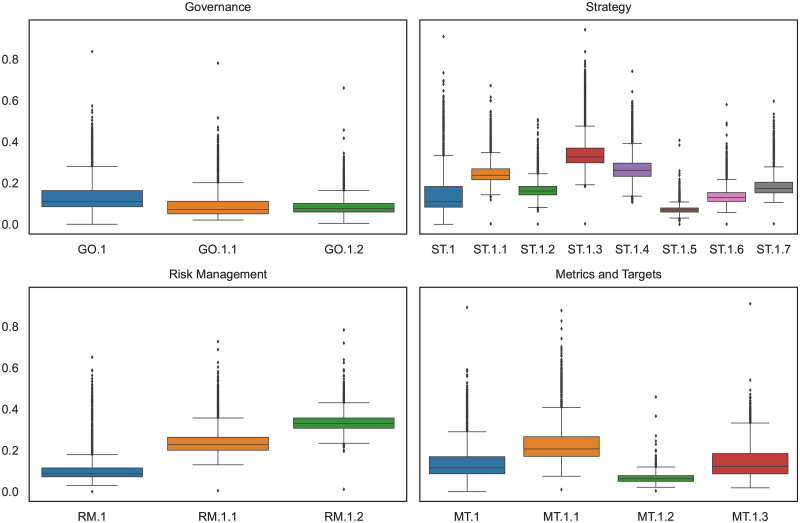
Climate-related disclosures by fine-grained TCFD labels.

Fig 4 displays the results of the zero-shot text classification for the fine-grained labels applied to the entire sample of reports. As can be seen, there is considerable variation in the extent of disclosure within each TCFD category. The strategy category is the most comprehensive, encompassing several specific recommended disclosures for banks, which explains the presence of a greater number of labels compared to the other pillars. The reporting quality appears to be lower for disclosures related to financing and investment in carbon-intensive industries such as the fossil fuel industry (ST.1.5), as indicated by a probability of only 7%. This suggests that among all the text sequences extracted from our full sample of reports and classified by the zero-shot model, there is only a 7% probability of some of them matching the semantics of the ST.1.5 label. Similarly, TCFD-supporting banks seem to provide relatively limited disclosure on climate-related physical risks (ST.1.2) and the use of climate-related scenario models (ST.1.6), with both labels attaining probabilities of only 17% and 14%, respectively.

There could be several interpretations for these results. First, it is possible that the reports in our sample only address to a limited extent issues linked to the use of scenario analyses or climate-related physical risks. This may be because some of the TCFD-supporting banks are still in the early stages of developing the necessary tools and expertise to conduct such analyses or identify such risks. This finding aligns with the study conducted by Bingler et al. [6], which highlights that only 10 out of 16 existing climate scenario tools allow for the assessment of climate-related physical risks. Similarly, Friederich et al. [2] find that disclosures on transition risks have seen more significant growth compared to disclosures on physical risks.

The low label probability for disclosures related to the fossil fuel industry (ST.1.5), indicates that banks tend to provide limited information on this topic. Nevertheless, recent research highlights that financing for fossil fuel firms by international banks has not decreased since the Paris Agreement, and these banks continue to provide funding regardless of the associated stranded asset risk [11]. In fact, Beyene et al. [11] specifically identified several TCFD-member banks, including JP Morgan (TCFD-member since December 2017), BNP Paribas SA (TCFD-member since June 2017) and Wells Fargo & Co (TCFD-member since November 2019), as among the top lenders to fossil fuel firms between 2007 and 2018. This suggests that TCFD-supporting banks may engage in selective disclosure, as suggested by Bingler et al. [6], potentially omitting certain information due to both reputational concerns and the absence of specific guidelines for measuring such exposures [3]. On the other hand, it is worth noting that banks appear to disclose more information regarding their credit exposure to carbon-related sectors. This is not surprising since this category encompasses a broader definition that includes sectors like transportation and utilities.

In the risk management area, we observe that banks tend to address processes for identifying, assessing, and managing climate-related risks and integrating them into overall risk management (RM.1.1) less frequently, on average, compared to the relationship between climate-related risks and financial risks (RM.1.2). The median value for RM.1.1 is also lower (23%) than in the case of RM.1.2 (33%). However, the probabilities provided by the zero-shot text classification could be slightly inflated compared to the actual reporting since the zero-shot performed less well for RM.1.2 (F1-score: 0.39). As shown previously, several sentence groups achieved a high probability of fitting into RM.1.1 and RM.1.2. Notably, several recommended disclosures within other pillars, such as the role of management in assessing and managing climate-related issues (GO.1.2), are also related to risk management topics.

In the metrics and targets category, we find that the incorporation of climate-related performance metrics into remuneration policies (MT.1.2) is associated with a lower label probability compared to metrics related to carbon footprints (MT.1.1) and emissions reduction targets (MT.1.3). The mean label probability for MT.1.2 is only 7%, while it is 24% for MT.1.1 and 15% for MT.1.3. This result aligns with our expectations, as financial institutions are less likely to align their compensation policies with climate-related performance metrics compared to making symbolic commitments to carbon neutrality goals, even if they may fail to meet these commitments (see e.g., [25, 26]).

In the governance area, the TCFD-supporting banks appear to report at comparable levels on the board’s responsibility for overseeing climate-related issues (GO.1.1) and the management’s role in assessing and managing climate-related issues (GO.1.2). Both labels exhibit a relatively low average probability of 9%, with median values of approximately 7% for GO.1.1 and 8% for GO.1.2. However, the maximum values for GO.1.1 are higher, indicating more comprehensive disclosures on the role of the board in overseeing climate-related issues.

In order to better assess the evolution of reporting on the underlying recommended disclosures, we report in Table 8 the mean label probabilities of the fine-grained labels in each financial year. We observe that an increase in mean label probabilities can be observed for most labels between 2017 and 2018, and in particular between 2018 and 2019.

**Table 8:** Mean of label probabilities for fine grained labels per financial year.

	2010	2011	2012	2013	2014	2015	2016	2017	2018	2019	2020	2021
GO.1.1	0,07	0,07	0,08	0,07	0,07	0,08	0,08	0,08	0,09	0,11	0,13	0,14
GO.1.2	0,08	0,07	0,08	0,07	0,08	0,08	0,08	0,08	0,09	0,10	0,11	0,12
ST.1.1	0,24	0,24	0,24	0,23	0,24	0,24	0,24	0,24	0,25	0,26	0,28	0,28
ST.1.2	0,16	0,16	0,16	0,16	0,16	0,16	0,16	0,16	0,17	0,18	0,19	0,19
ST.1.3	0,34	0,34	0,34	0,33	0,33	0,34	0,33	0,33	0,34	0,36	0,40	0,40
ST.1.4	0,25	0,25	0,26	0,25	0,26	0,26	0,27	0,26	0,27	0,28	0,31	0,32
ST.1.5	0,07	0,07	0,07	0,07	0,07	0,07	0,07	0,07	0,07	0,07	0,08	0,08
ST.1.6	0,13	0,13	0,13	0,12	0,13	0,13	0,13	0,13	0,14	0,15	0,16	0,16
ST.1.7	0,18	0,17	0,18	0,17	0,17	0,18	0,18	0,18	0,19	0,20	0,22	0,22
RM.1.1	0,22	0,22	0,23	0,22	0,23	0,23	0,23	0,23	0,24	0,25	0,27	0,28
RM.1.2	0,34	0,34	0,34	0,33	0,33	0,33	0,33	0,32	0,33	0,34	0,35	0,35
MT.1.1	0,21	0,21	0,21	0,21	0,21	0,22	0,22	0,22	0,23	0,25	0,28	0,29
MT.1.2	0,06	0,06	0,06	0,06	0,06	0,06	0,06	0,07	0,07	0,07	0,07	0,07
MT.1.3	0,12	0,12	0,13	0,12	0,13	0,13	0,14	0,14	0,15	0,16	0,18	0,19

The table presents the descriptive statistics of label probabilities (on a scale from 0 to 1) for the fine-grained TCFD labels based on the full sample of 3,355 reports.

### Climate-related disclosures after individual TCFD support

In the following step, we examine whether the observed increase in climate-related disclosures following the introduction of the TCFD recommendations is statistically significant for both the overall fine-grained labels and after individual banks declared their support. As the process of joining the official TCFD supporters was gradual, with banks joining at different times, we can investigate whether climate-related disclosures truly increased after banks individually began supporting the TCFD recommendations. Our approach builds upon the study of Bingler et al. [6], but extends the analysis by focusing on the specific recommended disclosure topics rather than the broad category level.

Table 9 presents the results of a paired t-test (in percentage points), in which we compare the mean differences of label probabilities before and after the official TCFD introduction in 2017 (column 1), as well as before and after the year of individual TCFD support (columns 2 to 6). As a robustness check, we also performed permutation p-value tests using a Monte Carlo simulation and bootstrap confidence intervals, which yielded qualitatively similar results. As an illustration, if banks became TCFD supporters in 2018, we compare the mean of each label probability for all of these banks (26 in total) by taking the mean per bank from 2010 to 2017 and comparing it to the mean from 2018 to 2021 after the banks became supporters. For the full sample, in column 1, we compare the mean difference up to the publication of the official TCFD recommendations (mean of years 2010 to 2016) and after they were published (mean of years 2017 to 2021).

**Table 9:** Mean differences in percentage points of climate-related disclosures.

	Full Sample	TCFD support since				
		2017	2018	2019	2020	2021
	n = 188	n = 38	n = 26	n = 25	n = 30	n = 53
	(1)	(2)	(3)	(4)	(5)	(6)
GO.1	3.59***	5.69***	4.72***	4.25***	4.51***	3.24***
GO.1.1	3.29***	5.02***	4.43***	3.74***	4.34***	3.12***
GO.1.2	2.21***	3.34***	2.88***	2.61***	2.98***	2.25***
ST.1	4.80***	7.70***	6.51***	5.61***	6.16***	4.23***
ST.1.1	2.41***	4.15***	3.41***	2.59***	3.22***	2.54***
ST.1.2	1.47***	2.57***	1.89***	1.80***	2.10***	1.75***
ST.1.3	2.74***	4.84***	5.36***	4.05***	4.52***	3.28***
ST.1.4	2.45***	4.60***	3.98***	2.53***	3.25***	3.10***
ST.1.5	0.50***	1.19***	0.96***	0.28	0.65***	0.71**
ST.1.6	1.59***	3.24***	2.19***	1.77***	1.75***	1.93***
ST.1.7	2.44***	4.13***	3.54***	3.02***	3.24***	2.45***
RM.1	3.39***	5.76***	5.02***	3.42***	4.25***	3.09***
RM.1.1	2.82***	4.72***	3.84***	3.11***	3.56***	2.74***
RM.1.2	0.33*	1.12**	2.20***	0.20	1.40**	1.29**
MT.1	3.90***	6.22***	5.03***	4.57***	4.78***	3.62***
MT.1.1	4.06***	6.43***	5.44***	5.28***	5.46***	3.75***
MT.1.2	0.63***	1.06***	0.37*	1.03***	0.55**	0.86**
MT.1.3	3.74***	5.34***	4.19***	4.94***	4.56***	3.38***

Note: *p < 0.1;

**p < 0.05;

***p < 0.01.

This table presents the mean differences (in percentage points) of climate-related disclosures and the significance of the corresponding paired t-Test based on the full sample of 3,355 reports.

In column 1, we observe a small but statistically significant increase in climate-related reporting probabilities for the full sample following the official introduction of the TCFD in 2017. The mean differences are statistically significant at the 1% level, except for RM 1.2, which is significant at the 10% level. On average, we find a total increase of 2.72 percentage points across all labels, which aligns with the findings of Bingler et al. [6], who report an increase of approximately 2.2 percentage points. In columns 2 and 3, we report that the groups of banks that became supporters in 2017 and 2018 exhibit higher disclosure levels after their individual support, as illustrated by the relatively larger and statistically significant mean differences. For the group of banks who became official supporters in 2019, as shown in column 4, we do not find any significant change in mean label probabilities for two labels. This result is consistent with Bingler et al. [6] who report the largest nominal effects for companies that supported the TCFD recommendations directly in 2017 and 2018 as compared to companies that supported the TCFD in 2019 and 2020.

In terms of magnitude, the greatest differences in reporting probabilities for banks that joined the TCFD in 2017 are found in the fine-grained labels MT.1.1 (carbon footprint, 6.43%), MT.1.3 (emissions reduction and carbon neutrality targets, 5.34%), and GO.1.1 (board oversight of climate-related issues, 5.02%). For the banks that joined in 2018, we find the largest mean differences for the labels MT.1.1 (carbon footprint, 5.44%), ST.1.3 (financial impact of climate-related issues, 5.36%) and GO.1.1 (board oversight of climate-related issues 4.43%). Similarly, for banks that joined in 2019, 2020, and 2021, the largest differences are found in the labels MT.1.1 (carbon footprint), MT.1.3 (emissions reduction and carbon neutrality targets), and ST.1.3 (financial impact of climate-related issues).

Altogether, the largest differences tend to be oftentimes observed in the Metrics and Targets category and pertain to carbon footprints as well as emissions reduction targets. Thus, TCFD-supporting banks appear to increase their level of disclosures related to these topics in the course of their official TCFD endorsement. One possible reason for this observation could be that the importance that stakeholders, including investors, place on carbon risk [26]. This is also consistent with Ding et al. [9] who show that there is a positive relationship between carbon emissions and climate-related disclosures.

### Climate-related disclosures by bank size

Finally, we examine the relationship between bank size and climate-related reporting, taking into consideration that larger firms generally have more resources and stronger corporate social responsibility (CSR) profiles [14]. We anticipate that larger banks are more likely to produce climate-related reports that align with the TCFD recommendations, resulting in higher label probabilities compared to medium and small banks.

To examine TCFD reporting based on bank size, we employ a Tukey test, assessing the differences between the means of the different bank sizes. Table 10 summarizes the results of the Tukey test for the differences between the means and their adjusted p-values corrected for family-wise error rate. Consistent with our expectations, we report that mean scores for all TCFD recommendations significantly increase from medium to large banks and from small to large banks, suggesting that larger banks generally are more likely to produce reports that align with the TCFD recommendations. In contrast, the effects are nominally smaller between small and medium banks compared to the other groups.

**Table 10:** Tukey difference-in-mean test of climate-related disclosures.

	Large—Medium	Large—Small	Medium—Small
GO.1	2.06***	3.46***	1.40***
GO.1.1	1.24***	2.23***	0.99***
GO.1.2	0.93***	1.58***	0.65***
ST.1	2.55***	4.68***	2.13***
ST.1.1	1.64***	2.35***	0.71**
ST.1.2	0.95***	0.96***	0.01
ST.1.3	3.06***	4.15***	1.09**
ST.1.4	2.67***	2.83***	0.16
ST.1.5	0.73***	0.71***	0.01
ST.1.6	1.76***	1.69***	0.07
ST.1.7	1.48***	2.30***	0.83***
RM.1	2.03***	2.91***	0.88***
RM.1.1	1.87***	2.40***	0.54
RM.1.2	1.40***	1.93***	0.54*
MT.1	2.38***	3.83***	1.46***
MT.1.1	2.39***	3.86***	1.48***
MT.1.2	0.46***	0.67***	0.21
MT.1.3	1.72***	3.46***	1.74***

Note: *p < 0.1;

**p < 0.05;

***p < 0.01.

This table presents the mean differences (in percentage points) of climate-related disclosures and the significance of the corresponding Tukey difference-in-mean test based on the full sample of 3,355 reports.

In terms of magnitude of the results, we also observe differences across the labels. Notably, we find that the largest differences between large and small banks relate primarily to reporting on the financial impact of climate-related issues (ST.1.3), carbon footprint (MT.1.1) and emissions reduction targets (MT.1.3). These findings support our initial expectations, as larger banks possess greater capacities to measure and report their carbon footprints. Furthermore, they are more likely to consider the material financial impacts of climate change, given their larger portfolios and potential exposure to transition or physical climate risks.

## Discussion and conclusion

This paper examines the climate-related disclosures of TCFD-supporting banks using the zero-shot text classification as a novel computerized approach for textual analysis of climate-related disclosures. By combining the TCFD recommendations with additional guidance specific to the financial sector, we create fine-grained labels that enable a more detailed examination of climate-related reporting. Our findings reveal significant variation in climate-related disclosures, not only across the broad TCFD categories but also within each category. Specifically, we observe that banks have a lower probability of reporting on topics such as climate-related physical risks, financing and investments in fossil fuel activities, the use of climate-related scenario models, and the integration of climate-related performance metrics into remuneration policies. These results indicate that the TCFD-supporting banks in our sample have not yet implemented all the recommendations to the same extent. Our research contributes to the expanding body of literature on voluntary climate-related corporate reporting (e.g., [2, 6, 9]).

Our study also entails some limitations, which warrant careful consideration and indicate potential areas for future research. First, although we observe an overall increase in climate-related reporting following the release of the TCFD recommendations, it is important to note that this does not necessarily imply that banks are taking more substantial internal actions to address the identified issues. Simply disclosing more information does not guarantee a corresponding increase in efforts to address climate-related challenges. This highlights the need for further research regarding the factors influencing reporting decisions. Additionally, there is a possibility that some banks intentionally do not disclose certain information and engage in selective disclosure (i.e., greenwashing) to improve their public image. This emphasizes the need to consider potential motivations and biases behind the disclosed information in the context of voluntary disclosures. Addressing these limitations and exploring these areas in future research can contribute to a deeper understanding of the relationship between climate-related reporting, internal actions, and the effectiveness of voluntary disclosure frameworks such as the TCFD. Furthermore, our research also reveals weaknesses in the TCFD recommendations, such as a lack of precise concepts and overlaps in recommended disclosures. Future research could therefore further investigate the determinants of high-quality climate-related disclosure frameworks and assess their impact in delivering material and decision-useful information.

Despite the limitations mentioned, our study has important practical implications. It underscores the necessity for precise and specific recommendations within climate-related disclosure frameworks. Without such consistent methodologies and explicit definitions for recommended disclosure topics, there is a potential for significant variation in the scope and depth of reporting. In the banking sector, while the TCFD recommendations are a positive step forward, the lack of concrete guidance hinders accurate assessment of banks’ exposure to the fossil fuel sector and potential stranded assets. Addressing these issues can enhance the effectiveness and reliability of climate-related reporting frameworks, facilitating informed decision-making by stakeholders.

## Supporting information

S1 Data Dataset underlying the results described in this manuscript. (CSV)
